# Outcome and toxicity of ifosfamide, carboplatin, and etoposide versus gemcitabine and vinorelbine regimen for pediatric patients with relapsed or refractory Hodgkin’s lymphoma

**DOI:** 10.3389/fonc.2023.1153128

**Published:** 2023-06-27

**Authors:** Ahmed Mahdy, Asmaa Hamoda, Ahmed Zaher, Eman Khorshed, Madeha Elwakeel, Omneya Hassanein, Iman Sidhom

**Affiliations:** ^1^ Pediatric Oncology Department, Children's Cancer Hospital Egypt, Cairo, Egypt; ^2^ Pediatric Oncology Department, National Cancer Institute, Cairo University, Cairo, Egypt; ^3^ Nuclear Medicine Department, National Cancer Institute, Cairo University, Cairo, Egypt; ^4^ Nuclear Medicine Department, Children's Cancer Hospital Egypt, Cairo, Egypt; ^5^ Surgical Pathology Department, National Cancer Institute, Cairo University, Cairo, Egypt; ^6^ Surgical Pathology Department, Children's Cancer Hospital Egypt, Cairo, Egypt; ^7^ Radiology Department, National Cancer Institute, Cairo University, Cairo, Egypt; ^8^ Radiology Department, Children's Cancer Hospital Egypt, Cairo, Egypt; ^9^ Clinical Research Department, Children's Cancer Hospital Egypt, Cairo, Egypt

**Keywords:** relapsed/refractory pediatric CHL, ICE, GV, toxicity, outcome

## Abstract

**Background:**

Pediatric classical Hodgkin lymphoma (CHL) is a curable disease; however, the optimal salvage regimen is unclear for relapsed/refractory (R/R) disease. This study aimed to compare response rates, toxicity, event-free survival (EFS), and overall survival (OS) of ifosfamide, carboplatin, and etoposide (ICE) with gemcitabine and vinorelbine (GV) regimen after first-line doxorubicin, bleomycin, vinblastine, dacarbazine (ABVD) in pediatric patients with R/R CHL.

**Methods:**

This is a retrospective cohort study of 132 pediatric patients with R/R CHL treated from July 2012 to December 2020 with ICE (n = 82) or GV (n = 50).

**Results:**

The median age at relapse was 13.9 years, and 68.2% were men. Rates of complete response, partial response, and progressive disease before consolidation were 50.6%, 3.7%, and 45.7% for ICE and 28.5%, 0%, and 71.5% for GV (P = 0.011). By multivariate analysis, regimen (P = 0.002), time to relapse (P = 0.0001), and B-symptoms (P = 0.002) were independent factors to lower response rates. Hematological toxicity, electrolyte disturbance, hemorrhagic cystitis, infectious complications, and hospital admission for fever neutropenia were statistically significant higher for the ICE regimen. Treatment-related mortalities were 2.4% for ICE and 2% for GV (P = 0.86). The 3-year EFS was 39.3% ± 11.4% for ICE and 24.9% ± 12.5% for GV (P = 0.0001), while 3-year OS was 69.3% ± 10.6% and 74% ± 12.9% (P = 0.3), respectively. By multivariate analysis, regimen (P = 0.0001), time to relapse (P = 0.011), B-symptoms (P = 0.001), and leukocytosis (P = 0.007) were significant for EFS, while anemia (P = 0.008), and progressive disease on early response evaluation (P = 0.022) were significant for OS.

**Conclusions:**

The ICE regimen had a better overall response rate and EFS, but higher toxicity, than GV; however, OS and mortality were similar.

## 1 Introduction

Relapsed or refractory (R/R) classical Hodgkin lymphoma (CHL) remains a clinical and therapeutic challenge. Approximately 10%–30% of patients relapse after first-line chemotherapy (1–3). The possibility of a cure after relapse depends on several prognostic factors, including duration of initial remission, stage, B-symptoms, and response to standard dose salvage chemotherapy (SDCT) (4–8). However, the duration of initial remission and response to pre-transplant SDCT remains the most important prognostic factors (7–11). The addition of brentuximab vedotin to upfront standard chemotherapy for newly diagnosed high-risk patients or those with R/R disease resulted in higher efficacy, without an increase in toxicity (12–14). Also, the combinations of brentuximab vedotin plus nivolumab, pembrolizumab, or nivolumab with conventional chemotherapy resulted in higher complete response rates and successful bridging to autologous stem cell transplant (ASCT) (15–17). However, the optimal pre-transplant SDCT for R/R CHL patients is unclear and needs more studies, as there are few studies in children addressing the question of what is the ‘best’ SDCT in terms of high efficacy and low toxicity (18). Therefore, we aimed to compare the outcome and short-term toxicities of an ifosfamide, carboplatin, and etoposide (ICE) regimen, with gemcitabine and vinorelbine (GV) in patients with R/R CHL.

## 2 Patients and methods

This retrospective study included all pediatric patients, <18 years at initial diagnosis, with R/R CHL who received SDCT either an ICE or GV regimen after the first-line doxorubicin, bleomycin, vinblastine, and dacarbazine (ABVD) in the pediatric oncology department, Children Cancer Hospital Egypt, from July 2012 to December 2020. All patients were followed until December 2021.

Patients with nodular lymphocyte–predominant HL (19) and patients with immunodeficiency were excluded as they were treated initially on different protocols other than the ABVD regimen. All the information concerning clinicopathological characteristics, time to relapse, response evaluations, toxicities, and survival were collected from the electronic medical records. The study was approved by the institutional review board.

### 2.1 Primary therapy

The ABVD regimen was administered intravenous (IV) on days 1 and 15 as follows: vinblastine, 6 mg/m^2^; bleomycin, 10 international unit/m²; doxorubicin, 25 mg/m^2^; and dacarbazine, 375 mg/m^2^.

### 2.2 Salvage therapy

A total of 82 and 50 patients received ICE and GV regimens, respectively. ICE was the second-line SDCT for all relapsed or refractory cases from July 2012 to September 2017; then, the salvage was changed to the GV regimen. Treatment courses are repeated every 3–4 weeks upon hematological recovery for at least two courses in the absence of disease progression. The ICE regimen was administered as follows: ifosfamide, 1,800 mg/m^2^/day with an equal dose of MESNA (2-mercaptoethane sulfonate), IV on days 1–5; carboplatin 450 mg/m^2^/day IV on day 1; and etoposide, 100 mg/m^2^/day IV on days 1–5. The GV regimen was administered IV on days 1 and 8 as follows: vinorelbine, 25 mg/m^2^; and gemcitabine 1000 mg/m^2^. CMV (carboplatin, melphalan, and etoposide was the preparative regimen before ASCT before February 2017 and then changed to bendamustine, etoposide, cytarabine, and melphalan (BEAM) thereafter.

### 2.3 Definitions

#### 2.3.1 Relapse criteria

Based on time to relapse after ABVD therapy, patients were categorized into three groups; refractory defined as disease progression or relapse within 3 months after ABVD therapy, early relapse as a CR lasting for ≥3 months but <12 months from ABVD therapy, and late relapse as CR ≥12 months (1) (7) (20). Relapse was documented by tissue biopsy in 115 (87%) patients. In emergency situations necessitating urgent initiation of chemotherapy, or failure to document by tissue biopsy, unequivocal progressive or new radiological lesions were accepted as proof of relapse in the absence of another possible explanation.

#### 2.3.2 Staging

All patients were staged according to the Ann Arbor staging system and underwent fluorodeoxyglucose positron emission tomography (FDG-PET) scan and bilateral bone marrow biopsies (BMB).

#### 2.3.3 B-symptoms

Patients with fevers ≥ (38.3°C), drenching night sweats, or unexplained weight loss > 10% of body mass (21) are defined to have B symptoms.

#### 2.3.4 Tumor bulk

A single nodal mass of 10 cm or ≥⅓ of the transthoracic diameter at any level of thoracic vertebrae, recording the longest measurement by computed tomography (CT) scan (21).

#### 2.3/5 Response definition

Response to SDCT was decided based on PET/CT scans and BMB, if positive at relapse, after two to three courses (early response), fourth course, and before consolidation as an overall response rate (ORR) according to the standard response definition criteria by the Lugano Classification 2014 (21) using the 5-point scale and are defined as achieving a complete response (CR), partial response (PR), stable disease (SD), or progressive disease (PD). Patients with PR or SD continued to receive SDCT for a maximum of six cycles or till consolidation with ASCT or radiotherapy, which comes earlier. Patients with PD at any time were off protocol study.

### 2.4 Toxicities

Grade III–IV acute toxicities and toxic death with a time frame of up to 4 weeks following each course of chemotherapy were collected and compared between both regimens according to the National Cancer Institute Common Terminology Criteria for Adverse Events (CTCAE) v5.0 (22).

### 2.5 Prognostic factor analysis

A spectrum of prognostic factors at relapse were evaluated for significance to the second-line ORR, event-free survival (EFS), and overall survival (OS) including the following; sex, age (<13 vs. ≥13 years), stage, histology, B-symptoms, bulky disease, leukocyte count (<13.5 vs. ≥13.5 × 10^3^/mm^3^), hemoglobin (<10.5 vs. ≥10.5 gm/dl), erythrocyte sedimentation rate (ESR; <50 vs. ≥50 mm/h), lactate dehydrogenase (LDH; <700 vs. ≥700 IntUnit/L), albumin (<3.5 vs. ≥3.5 gm/dl), radiotherapy during first line, time to relapse, early response, and overall response. Cutoff values used to categorize these factors were based on values found to be predictive of outcome in previous studies (9) (23) (24).

### 2.6 Statistical analysis

The endpoints analyzed were early response after two to three cycles, ORR, EFS, and OS. EFS was calculated from the date of start of SDCT until the date of progression, death, or last follow-up, and OS from the date of diagnosis until the date of death or last follow-up. EFS and OS were estimated using the Kaplan–Meier method (25). Univariate analyses of clinicopathological characteristics were performed to select covariates for multivariate analyses. The influence of these factors on the OS and EFS was estimated by Cox proportional hazards regression (26). The hazard ratio was calculated for each variable with 95% confidence intervals (95% CIs). All factors with a P-value ≤ 0.10 in univariate analysis were subject to stepwise Cox regression analysis. Effect of factors on the ORR was estimated using logistic regression. Numerical values were summarized using median and range. Categorical variables expressed as the number of cases and percentages, and groups were compared using the chi-square or Fisher’s exact test as appropriate. Analyses were performed using BM-SPSS Statistics 25.

## 3 Results

### 3.1 Patient characteristics

As shown in **Table 1**, the median age at relapse or progression was 13.9 years (range, 3–24 years) and 68.2% were men. Clinicopathological characteristics were quite similar in the two protocols, except 47.6% of the ICE group did not receive radiotherapy versus 30% of the GV (P = 0.047). Furthermore, there was a trend of higher lung involvement in the ICE (29.3%), versus the GV group (16%), but it did not reach statistical significance (P = 0.06).

**Table 1:** Patients and disease characteristics at first relapse.

Characteristic	ICE Group Number = 82 (%)	GV Group Number = 50 (%)	P-value
Sex			
Male	58 (70.7)	32 (64)	0.42
Female	24 (29.3)	18 (36)	
Age (years)			
<13	43 (52.4)	19 (38)	0.1
≥13	39 (47.6)	31 (62)	
Histology			
Nodular sclerosis	44 (62.8)	32 (66.7)	0.75
Others	26 (37.2)	16 (33.3)	
Missing	12 (14.6)	2 (4)	
Time to relapse			
Refractory	19 (23.2)	12 (24)	0.88
Early relapse	16 (19.5)	8 (16)	
Late relapse	47 (57.3)	30 (60)	
Stage			
I	9 (11)	3 (6)	0.87
II	12 (14.6)	10 (20)	
III	19 (23.2)	13 (26)	
IV	42 (51.2)	24 (48)	
Number of organs involved in stage 4			
1	23 (54.8)	14 (58.3)	0.12
2	11 (26.2)	10 (41.7)	
3	6 (14.2)	0 (0)	
4	2 (4.8)	0 (0)	
Bone marrow involvement~			
Present	15 (18.3)	12 (24)	0.4
Absent	67 (81.7)	38 (76)	
Bone involvement			
Present	18 (22)	10 (20)	0.9
Absent	64 (78)	40 (80)	
Liver involvement			
Present	14 (17)	4 (8)	0.14
Absent	68 (83)	46 (92)	
Lung involvement			
Present	24 (29.3)	8 (16)	0.06
Absent	58 (70.7)	42 (84)	
B-symptoms			
Present	27 (45)	16 (59.3)	0.2
Absent	33 (55)	11 (40.7)	
Missing	22 (26.8)	27 (54)	
Bulky disease			
Present	11 (13.4)	7 (14)	0.9
Absent	71 (86.6)	43 (86)	
WBC × 10^(3)^/mcl			
<13.5	63 (76.8)	34 (68)	0.27
≥13.5	19 (23.2)	16 (32)	
Hemoglobin, gm/dl			
<10.5	40 (48.8)	23 (46)	0.76
≥10.5	42 (51.2)	27 (54)	
ESR (first hour), mm/h			
<50	29 (40.3)	18 (38.3)	0.83
≥50	43 (59.7)	29 (61.7)	
Missing	10 (12.2)	3 (6)	
LDH, IntUnit/L*			
<700	56 (82.4)	42 (89.4)	0.3
≥700	12 (17.6)	5 (10.6)	
Missing	14 (17)	3 (6)	
Albumin, gm/dl			
<3.5	27 (64.3)	4 (36.4)	0.09
≥3.5	15 (35.7)	7 (63.6)	
Missing	40 (48.8)	39 (78)	
Radiation therapy (first line)			
No	39 (47.6)	15 (30)	0.047
Yes	43 (52.4)	35 (70)	

ICE, ifosfamide, carboplatin, and etoposide; GV, gemcitabine and vinorelbine; WBC, white blood cell; ESR, erythrocyte sedimentation rate; LDH, lactate dehydrogenase. ~Bone Marrow Involvement by BMB and/or PET/CT. ^*^LDH ≥ 1 × Normal (700 IntUnit/L). Percentages (%) and the P-value calculated on valid data only.

### 3.2 Response rates

The median number of the ICE chemotherapy cycles was 4.74 (range, 2–6 cycles), and 4.7 cycles (range, 2–6 cycles) for the GV group. The early response could be evaluated in 131 (99.2%) and ORR before consolidation in 130 (98.5%) patients, as one died after the second-cycle ICE and one after the fourth^-^cycle GV. On early response evaluation, 54.3% of the ICE group achieved CR, 40.7% had PR, and 5% had PD versus 34%, 50%, and 16%, of the GV group, respectively (P = 0.024). Rates of CR, PR, and PD before consolidation were 50.6%, 3.7%, and 45.7%, respectively, for ICE and 28.5%, 0%, and 71.5%, respectively for GV (P = 0.011). There were 18 patients who achieved CR while receiving the ICE regimen but lost response and had PD. There were 12 of them who were found to have PD on routine evaluation after chemotherapy cycles and six patients on routine PET/CT assessment before ASCT with a mean of 127 days between the last ICE cycle and pre-transplant evaluation due to the waiting list for transplant. Eight patients of the GV regimen lost response and had PD after achieving CR. Six of them had PD on routine evaluation after chemotherapy cycles, and two patients had PD in pre-transplant assessment with a mean of 86.5 days between the last GV cycle and pre-transplant evaluation. Due to the higher remission status achieved by the ICE regimen, 46.3% of patients underwent ASCT as consolidation therapy after ICE chemotherapy versus 26% of the GV patients (P = 0.02).

As demonstrated in **Table 2**, by univariate analysis, the following factors were statistically significant for lower ORR before consolidation; the GV regimen (P = 0.004), female sex (P = 0.04), age ≥ 13 years (P = 0.01), refractory disease (P = 0.0001) on ABVD, higher stage (P = 0.01), lung involvement (P = 0.005), liver involvement (P = 0.01), B-symptoms (P=0.001), partial response on early evaluation, ESR ≥ 50, mm/h (P = 0.0001), hemoglobin < 10.5 gm/dl (P = 0.02), and leukocytes ≥ 13.5 × 10^(3)^/mcl (P = 0.01). By multivariate analysis, only regimen (P = 0.002), time to relapse (P = 0.002), and B-symptoms (P = 0.002) were statistically significant.

**Table 2:** Response rate for the whole cohort before consolidation in relation to prognostic factors.

Characteristic	Whole patients, number = 130 (%)				
	Response rate before consolidation, number (%)		Total	Univariate	Multivariate
	CR/PR	PD		P-value	P-value
Regimen					
ICE	44 (54.3)	37 (45.7)	81	0.004	0.002
GV	14 (28.6)	35 (71.4)	49		
Sex					
Female	13 (31.7)	28 (68.3)	41	0.04	NS
Male	45 (50.6)	44 (49.4)	89		
Age (year)					
<13	44 (53)	39 (47)	83	0.01	NS
≥13	14 (29.8)	33 (70.2)	47		
Time to relapse					0.002
Refractory	3 (10.3)	26 (89.7)	29	0.0001	0.0001
Early relapse	12 (50)	12 (50)	24		0.261
Late relapse (ref)	43 (55.8)	34 (44.2)	77		
Stage					
I	9 (75)	3 (25)	12	0.01	NS
II	13 (59.1)	9 (40.9)	22		
III	16 (50)	16 (50)	32		
IV	20 (31.3)	44 (68.7)	64		
Bone involvement					
Absent	48 (47.5)	53 (52.5)	101	0.2	NS
Present	10 (34.5)	19 (65.5)	29		
Bone marrow involvement ~					
Absent	49 (46.7)	56 (53.3)	105	0.34	NS
Present	9 (36)	16 (64)	25		
Lung involvement					
Absent	51 (51.5)	48 (48.5)	99	0.005	NS
Present	7 (22.6)	24 (77.4)	31		
Liver involvement					
Absent	55 (49.1)	57 (50.9)	112	0.01	NS
Present	3 (16.7)	15 (83.3)	18		
B-symptoms					
Absent	30 (68.2)	14 (31.8)	44	0.001	0.002
Present	13 (31.7)	28 (68.3)	41		
Bulky disease					
Absent	52 (46.4)	60 (53.6)	112	0.3	NS
Present	6 (33.3)	12 (66.7)	18		
Histology					
Nodular sclerosis	31 (41.9)	43 (58.1)	74	0.8	NS
Others	18 (43.9)	23 (56.1)	41		
ESR (first hour), mm/h					
<50	31 (66)	16 (34)	47	0.0001	NS
≥50	21 (30)	49 (70)	70		
WBC, ×10(3)/mcl					
<13.5	49 (51)	47 (49)	96	0.01	NS
≥13.5	9 (26.5)	25 (73.5)	34		
Hemoglobin, gm/dl					
<10.5	21 (33.9)	41 (66.1)	62	0.02	NS
≥10.5	37 (54.4)	31 (45.6)	68		
Albumin, gm/dl					
<3.5	14 (48.3)	15 (51.7)	29	0.7	NS
≥3.5	12 (54.5)	10 (45.5)	22		
LDH, IntUnit/L*					
<700	46 (47.4)	51 (52.6)	97	0.2	NS
≥700	5 (29.4)	12 (70.6)	17		
Early response					
CR	43 (70.5)	18 (29.5)	61	0.0001	NS
PR	15 (26.3)	42 (73.7)	57		
PD	0 (0)	12 (100)	12		

ICE, ifosfamide, carboplatin, and etoposide; GV, gemcitabine and vinorelbine; WBC, white blood cell; ESR, erythrocyte sedimentation rate; LDH, lactate dehydrogenase; CR, complete response; PR, partial response; PD, progressive disease; NS, not significant.

~Bone marrow involvement by bone marrow biopsy and/or PET/CT. ^*^LDH ≥ 1 × Normal (700 IntUnit/L).

**Figure 1:**
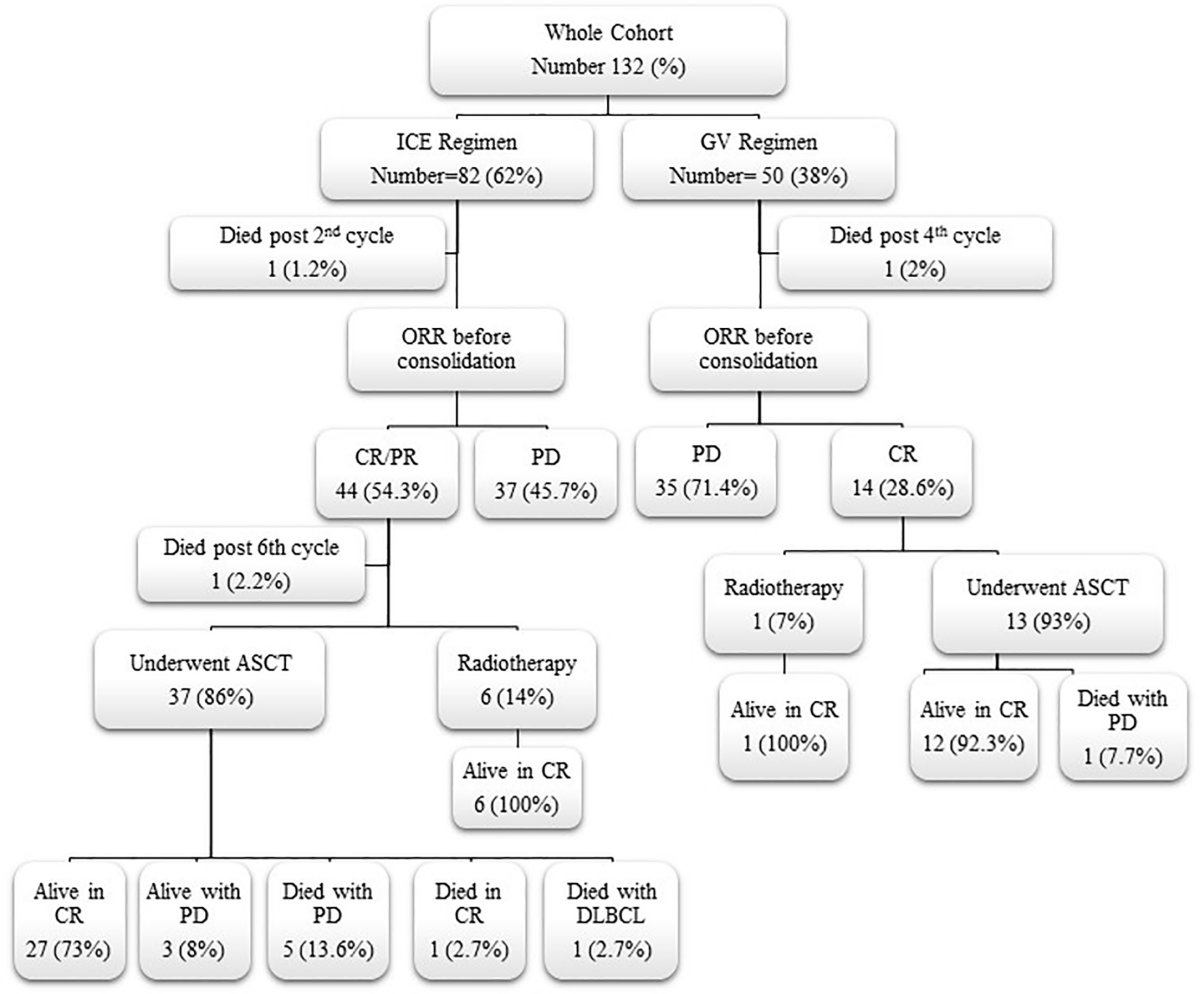
Flow chart for responding patients to second-line chemotherapy and their outcome. ICE, ifosfamide, carboplatin, and etoposide; GV, gemcitabine and vinorelbine; ORR, overall response rate; CR, complete response; PR, partial response; PD, progressive disease; ASCT, autologous stem cell transplantation; DLBCL, diffuse large B-cell lymphoma.

Consolidation therapy with ASCT or radiotherapy for responding patients and their outcome are shown in (**Figure 1**). For responding patients to ICE chemotherapy, the conditioning regimens used before stem cell rescue were CMV for 25 and BEAM for 13 patients, while 13 patients received BEAM, in the GV group. A total of 76% and 77% of patients maintained their CR after ASCT for the ICE, respectively, after CMV and BEAM versus 92% for the GV who received the BEAM preparative regimen.

### 3.3 Treatment and outcome for refractory disease to second line and second relapse

**Figure 2:**
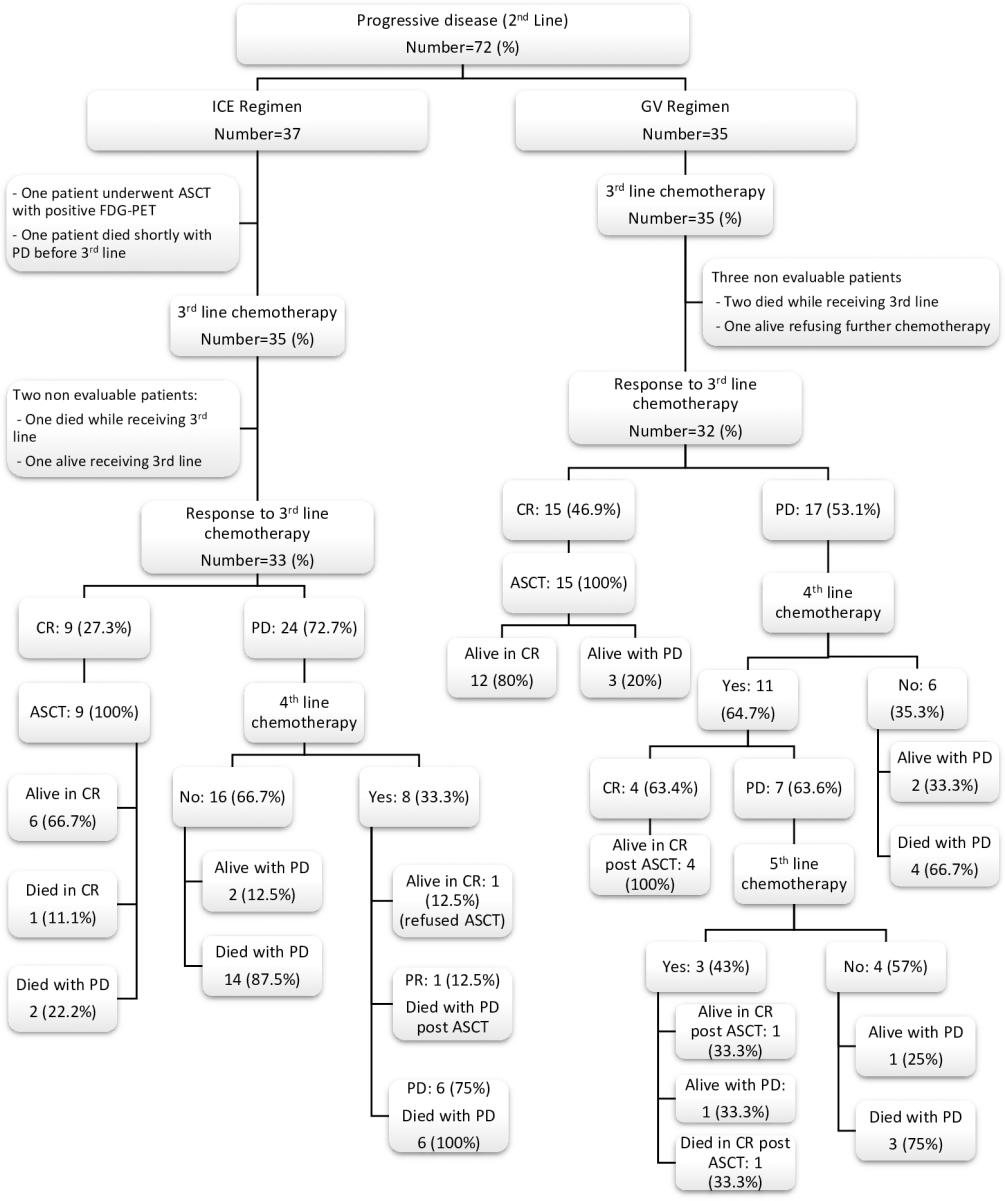
Flow chart for non-responding patients to second-line chemotherapy and their outcome after further salvage chemotherapy regimens. ICE, ifosfamide, carboplatin and etoposide; GV, gemcitabine and vinorelbine; CR, complete response; PR, partial response; PD, progressive disease; ASCT, autologous stem cell transplantation; FDG-PET, fluorodeoxyglucose (FDG)-positron emission tomography.

Thirty-five (70%) of the GV patients required salvage third-line chemotherapy after failure of the GV regimen versus 35 (42.7%) of the ICE regimen (P = 0.002). However, 34/35 (97%) patients of the GV group received the ICE regimen as a third line, and 15 of 32 evaluable patients achieved CR (46.9%). On the other hand, of the 35 patients who failed the ICE regimen, 27 of them received the GV as a salvage third-line chemotherapy, but only eight of them attained CR (29.6%). Again, it proves the superiority of the ICE regimen as salvage chemotherapy even as the third line in R/R HL patients. Eight patients of the ICE group received brentuximab vedotin with or without bendamustine during third or fourth lines, but only 2 patients responded versus 12 patients in the GV group with 50% of them achieving CR. Twenty-one (65.6%) patients of the GV group underwent ASCT post third, fourth, or fifth lines of chemotherapy versus 10 (30.3%) of the ICE group (P = 0.004). Detailed salvage chemotherapy regimens after second-line failure for both groups, response rates, ASCT, and survival are demonstrated in **Table 3** and **Figure 2**.

**Table 3:** Chemotherapy and response after second and multiple relapses.

Characteristic	ICE number (%)	GV number (%)	P-value
type of third line chemotherapy regimen	Number = 35	Number = 35	
ICE	0 (0)	34 (97.1)	–
Gemcitabine/Vinorelbine	27 (77.1)	0 (0)	
Brentuximab/Bendamustine	3 (8.6)	1 (2.9)	
Vinorelbine/Ifosfamide/Prednisone	3 (8.6)	0 (0)	
Bendamustine/Gemcitabine/Vinorelbine	1 (2.9)	0 (0)	
Ifosfamide/Gemcitabine/Vinorelbine/Predsol	1 (2.9)	0 (0)	
Response to third line before consolidation with ASCT	Number = 33	Number=32	
CR	9 (27.3)	15 (46.9)	0.10
PD	24 (72.7)	17 (53.1)	
Fourth-line salvage chemotherapy	Number=24	Number=17	
Yes	8 (33.3)	11 (64.7)	0.06
No	16 (66.7)	6 (35.3)	
Type of fourth-line Chemotherapy Regimen	Number=8	Number=11	
Brentuximab/Bendamustine	1 (12.5)	8 (72.7)	–
Brentuximab	4 (50)	0	
DHAP	3 (37.5)	3 (27.3)	
Response to fourth-line chemotherapy regimen	Number=8	Number=11	
CR	1 (12.5)	4 (36.4)	0.4
PR	1 (12.5)	0	
PD	6 (75)	7 (63.6)	
Type of fifth-line chemotherapy regimen	Number=0	Number=3	
Brentuximab/Bendamustine	0	2 (66.7)	–
Brentuximab/Nivolumab	0	1 (33.3)	
Response to fifth-line chemotherapy regimen	Number=0	Number=3	
CR	0	2 (66.7)	–
PD	0	1 (33.3)	
ASCT post third-, fourth-, or fifth-line chemotherapy	Number = 33	Number=32	
Yes	10 (30.3)	21 (65.6)	0.004
No	23 (69.7)	11 (34.4)	
Disease status post ASCT after multiple relapses	Number = 10	Number=21	
CR	7 (70)	18 (85.7)	0.3
PD	3 (30)	3 (14.3)	

ICE, ifosfamide, carboplatin, and etoposide; GV, gemcitabine and vinorelbine; ASCT, autologous stem-cell transplantation; DHAP, dexamethasone, cytarabine, cisplatin; CR, complete response; PR, partial response; PD, progressive disease.

For responding patients to further salvage regimens after ICE failure, the conditioning regimens used prior to stem cell rescue were CMV for six and BEAM for four patients. There were 21 patients who received BEAM, as conditioning regimens before ASCT for responding patients after GV failure. A total of 67% and 60% of patients maintained their CR after ASCT for the ICE, respectively, after CMV and BEAM versus 90% for the GV who received the BEAM preparative regimen.

### 3.4 Toxicity and mortality

The main acute toxicities of the ICE regimen were hematological. Of the 389 ICE cycles given, grade 3/4 neutropenia were documented in 96.6%, grade 3/4 thrombocytopenia in 82%, and grade 3 anemia in 69.4% of all cycles. Sixty percent of patients required platelet transfusions and 61.3% red blood cell (RBC) transfusions. Hospital admissions for supportive care with fever neutropenia (FN) and/or hemorrhagic cystitis were required in 54.2% of cycles. Clinically documented infections (CDIs), bloodstream infection (BSI), and septic shock were reported in 17.5%, 4.8%, and 5.6% of ICE cycles, respectively; however, no patients were removed from the ICE regimen on account of persistent toxicities. Only two (0.26%) treatment-related deaths were reported. One died of a diffuse alveolar hemorrhage after the second cycle and one of sepsis after the sixth cycle.

The GV was well tolerated with less acute toxicity compared to the ICE regimen. Of the 235 GV cycles given, grade 3/4 neutropenia was documented in 18.7%, grade 3/4 thrombocytopenia in 4.6%, and grade 3 anemia in 8% of all courses. Only 5.1% of patients required RBC transfusions and 0.8% platelet transfusions. Each of the hospital admissions with FN and CDI were reported in 2.1% of cycles. Grade 3–4 electrolytes disturbance, BSI, and septic shock were not reported. Only one treatment-related death was documented with pneumonia and respiratory failure after the fourth cycle. Seizures, cardiac, and hepatic toxicities were low and not statistically different between both groups. A comparison of the main toxicities between both regimens is summarized in **Table 4**.

**Table 4:** Comparison of mortalities and main toxicities between both regimens according to the Common Terminology Criteria for Adverse Events Version 5.0.

Mortality main toxicity	ICE	GV	p-value
	Total (389 cycles)	Total (235 cycles)	
	Number (%)	Number (%)	
Treatment-related mortality	2 (2.4)	1 (2)	0.861623
Hospital admission for supportive care	211 (54.2)	5 (2.1)	<0.0001
Hospital admission days (total all cycles)	2038	49	<0.0001
ICU admission	13 (3.4)	2 (0.8)	0.044688
Septic shock	22 (5.6)	0	<0.0001
WBC decreased (grade 3/4)	376 (96.6)	44 (18.7)	<0.0001
Neutrophil count decreased (grade 3/4)	372 (97.1)	91 (38.7)	<0.0001
Anemia (grade 3)*	270 (69.4)	19 (8)	<0.0001
Platelet count decreased (grade 3/4)	319 (82)	11 (4.6)	<0.0001
AST increased (grade 3)*	11 (2.8)	13 (5.5)	0.100801
ALT increased (grade 3)*	5 (1.2)	25 (10.6)	<0.0001
Total blood bilirubin increased (grade 3)*	6 (1.5)	2 (0.8)	0.438883
Hypokalemia (grade 3)*	112 (28.7)	0	<0.0001
Hypophosphatemia (grade 3)*	54 (13.4)	0	<0.0001
Hypomagnesemia (grade 3)*	102 (26.2)	0	<0.0001
Hemorrhagic cystitis (grade 3)*	31 (8)	0	<0.0001
Blood-stream infection	19 (4.8)	0	<0.0001
Clinically documented infection	68 (17.5)	5 (2.1)	<0.0001
Ejection fraction decreased (grade 3/4)	8 (2)	1 (0.4)	0.1638
Seizures (grade 3)*	2 (0.5)	0	0.527

ICE, ifosfamide, carboplatin, and etoposide; GV, gemcitabine and vinorelbine; ICU, intensive care unit; WBC, white blood cell; AST, aspartate transaminase; ALT, alanine aminotransferase. *No grade 4 toxicity reported.

### 3.5 Event-free survival and overall survival

**Figure 3:**
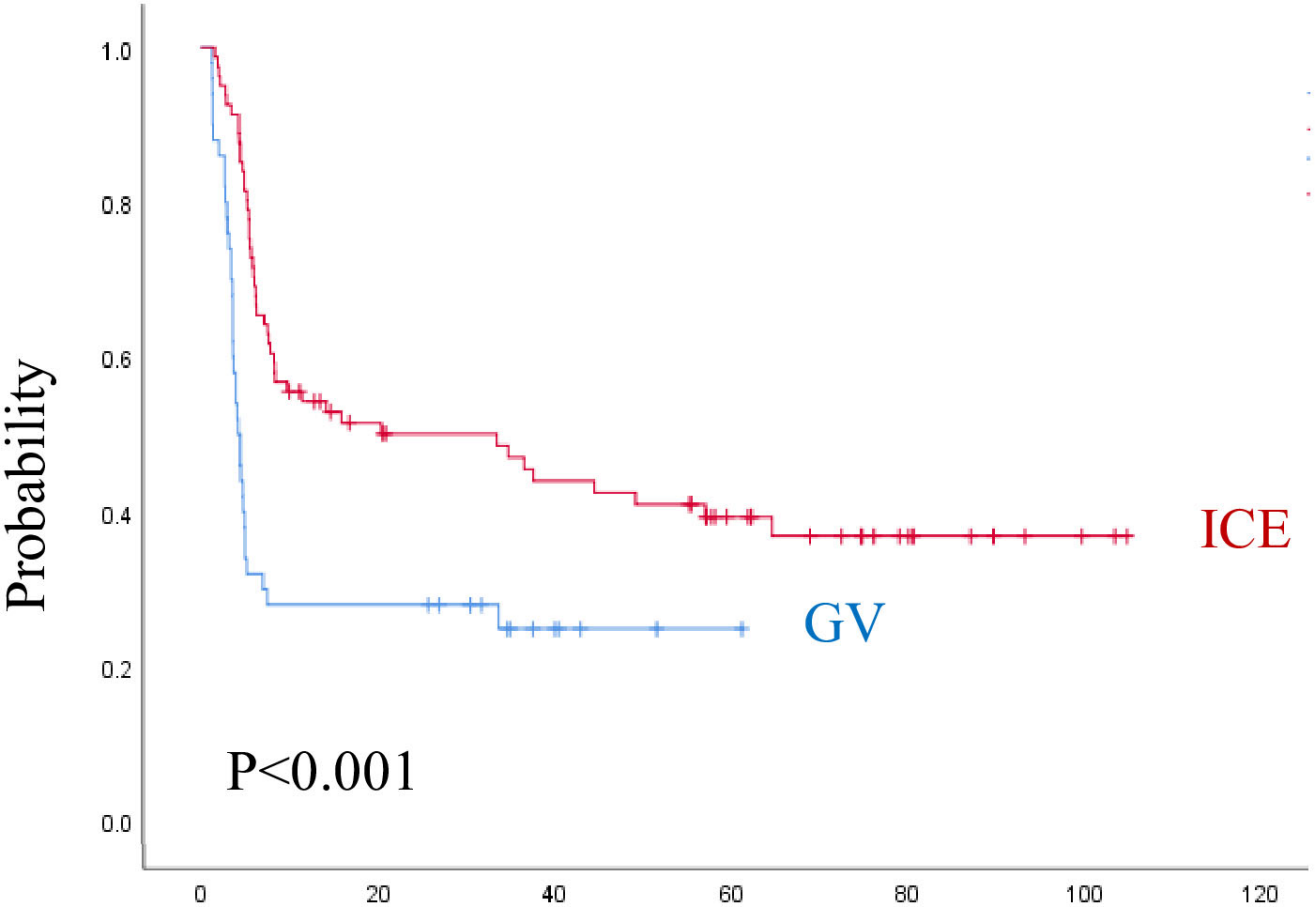
Event-free survival for both regimens. ICE, ifosfamide, carboplatin, and etoposide; GV, gemcitabine and vinorelbine.

**Figure 4:**
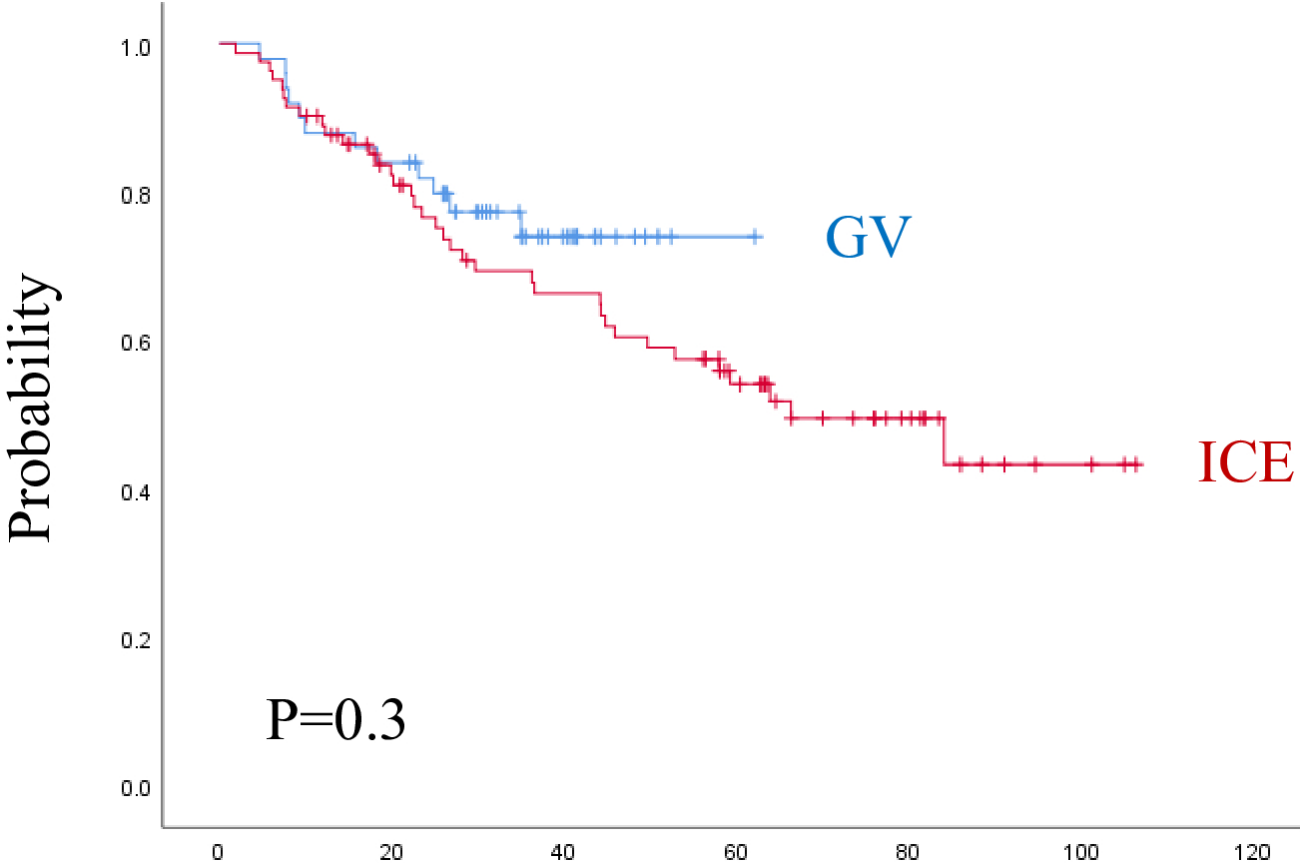
Overall survival for both regimens. ICE, ifosfamide, carboplatin, and etoposide; G: gemcitabine and vinorelbine.

The median follow-up time was 3.8 years (range, 0.1–8.8 years) for the ICE group and 2.6 years (range, 0.4–5.3 years) for the GV group. As shown in (**Figures 3**, **4**) and **Table 5**, the 3-year EFS was 39.3% ± 11.4% for the ICE regimen versus 24.9% ± 12.5% for the GV (P = 0.0001).

**Table 5:** Event-free survival and overall survival at 3 years in relation to prognostic factors.

Characteristics at relapse	3-Year EFS (%)	Univariate P-value	Multivariate P-value	3-Year OS (%)	Univariate P-value	Multivariate P-value
Regimen						
ICE	39.3 ± 11.4	0.001	0.0001	69.3 ± 10.6	0.3	NS
GV	24.9 ± 12.5			74 ± 12.9		
Sex						
Female	28.4 ± 13.8	0.03	NS	59.4 ± 15.5	0.02	NS
Male	43.5 ± 10.6			77.4 ± 9		
Age (year)						
<13	47 ± 11	0.01	NS	71.8 ± 9.8	0.4	NS
≥13	24.7 ± 12.3			68.3 ± 13.9		
Time to relapse			0.011			
Refractory	6.5 ± 8.6	0.001	0.008	47.4± 17.8	0.001	NS
Early relapse	40.7 ± 20		0.024	78.7 ± 16.7		
Late relapse (ref)	51 ± 11.6			79.6 ± 9.6		
Stage						
I	75 ± 24.5	0.008	NS	100	0.02	NS
II	53.4 ± 21.4			83.4 ± 17.4		
III	35.7 ± 17.2			76.3 ± 15.5		
IV	29.1 ± 11.2			60 ± 12.3		
Bone involvement						
Present	31.7 ± 17.4	0.5	NS	59.4 ± 18.6	0.4	NS
Absent	42 ± 9.6			74.9 ± 8.8		
Bone marrow involvement ~						
Present	28.8 ± 18.4	0.4	NS	67 ± 19	0.6	NS
Absent	39.9 ± 9.6			72.5 ± 8.8		
Liver involvement						
Present	17.6 ± 18	0.02	NS	37.8 ± 24.7	0.004	NS
Absent	41.9 ± 9.2			76.4 ± 8.2		
Lung involvement						
Present	18.8 ± 13.5	0.001	NS	44.9 ± 18.6	0.001	NS
Absent	45.2 ± 10			76.7 ± 8.8		
B-symptoms						
Present	28.7 ± 13.7	0.001	0.001	55.2 ± 15.7	0.03	NS
Absent	59.9 ± 14.5			82 ± 12.2		
Bulky disease						
Present	26.7 ± 21	0.13	NS	75 ± 22	0.7	NS
Absent	40.7 ± 9.2			70.8 ± 8.6		
Histology						
Nodular sclerosis	42.5 ± 14.9	0.8	NS	72.2 ± 10.4	0.76	NS
Others	47.7 ± 21			73.9 ± 14.11		
ESR (first hour), mm/h						
<50	58.5 ± 14.5	0.001	NS	83.8 ± 11.2	0.01	NS
≥50	25.1 ± 10.2			63 ± 11.4		
LDH, IntUnit/L^*^						
<700	41.3 ± 9.8	0.3	NS	75.5 ± 8.8	0.12	NS
≥700	23.4 ± 21.6			61.8 ± 24.2		
WBC, ×10^(3)^/mcl						
<13.5	46.1 ± 10	0.002	0.007	74.1 ± 9	0.5	NS
≥13.5	19.6± 13.3			31.2 ± 44.1		
Hemoglobin, gm/dl						
<10.5	28.6 ± 10.4	0.004	NS	62.1 ± 11.4	0.002	0.008
≥10.5	52.4 ± 13.5			84 ± 10.2		
Albumin, gm/dl						
<3.5	40 ± 17.4	0.58	NS	59.3 ± 17.8	0.1	NS
≥3.5	45.5 ± 20.8			77 ± 17.6		
Radiotherapy (first line)						
Yes	44.6 ± 11.6	0.08	0.025	63.7 ± 13.1	0.2	NS
No	30.1 ± 12.4			76.9 ± 10		
Early response						0.07
CR	62.7 ± 12.5	0.0001	NS	82.1 ± 10.2	0.0001	
PR	21.9 ± 11			40.4 ± 18		0.35
PD	0			41.7 ± 27.8		0.022

ICE, ifosfamide, carboplatin, and etoposide; GV, gemcitabine and vinorelbine; OS, overall survival; WBC, white blood cell; ESR, erythrocyte sedimentation rate; LDH, lactate dehydrogenase; CR, complete response; PR, partial response; PD, progressive disease; NS, not significant; ASCT, autologous stem cell transplantation. *LDH ≥ 1 × Normal (700 IntUnit/L).

When analyzing the prognostic factors by univariate analysis, regimen (P = 0.001), female sex (P = 0.03), age ≥ 13 years (P = 0.01), refractory disease during first line (P = 0.001), higher-stage (P = 0.008), liver involvement (P = 0.02), lung involvement (P = 0.001), B-symptoms (P = 0.001), ESR ≥ 50 mm/h (P = 0.001), leukocyte count ≥ 13.5 × 10^(3)^/mcl (P = 0.002), Hemoglobin < 10 gm/dl (P = 0.004), PD or PR on early evaluation (P = 0.0001) were statistically significant associated with lower EFS. By multivariate analysis, regimen (P = 0.0001), time to relapse (P = 0.011), B-symptoms (P = 0.001), leukocyte count ≥ 13.5 × 10^(3)^/mcl (P = 0.007), and radiotherapy during the first line (P = 0.025), were dominating over all other parameters. The 3-year OS was 69.3% ± 10.6% for the ICE regimen and 74% ± 12.9% for the GV (P = 0.3). The following factors were statistically significant for lower OS by univariate analysis, including, female sex (P = 0.02), RD during first-line (P = 0.001), higher-stage (P = 0.02), liver involvement (P = 0.004), lung involvement (P = 0.001), B-symptoms (P = 0.03), ESR ≥ 50 mm/h (P = 0.01), leukocyte count ≥ 13.5 × 10^(3)^/mcl (P = 0.002), hemoglobin < 10 gm/dl (P = 0.002), PD or PR at early evaluation (P = 0.0001), and PD before consolidation as ORR (P = 0.001). By multivariate analysis, only anemia (P = 0.008), and progressive disease on early response evaluation (P = 0.022) remain significant for OS.

The 3-year EFS for patients who underwent ASCT after second-line ICE was 79% ± 13.9% and 88.9% ± 20.6% after the second-line GV, while the 3-year EFS for patients who underwent ASCT after multiple lines in the ICE group was 71.4 ± 33.5% versus 70.5 ± 25.9% in the GV group.

## 4 Discussion

Our report is a single-center retrospective study on relapsed or refractory pediatric CHL after the ABVD regimen who received either the GV or the ICE regimen. Apart from fewer patients numbers in the GV group (n = 50) compared to the ICE (n = 82), both groups had relatively comparable clinicopathological characteristics and received two uniform salvage regimens; thus, it was possible to compare efficacy and outcome because of the homogeneity of patients’ characteristics and previous treatment received by both groups.

Our whole cohort revealed a male-to-female ratio of 2.2:1; this male predominance and other patients and disease characteristics at relapse were reported in many other studies (27–29). The percentage of patients with first treatment failure whether refractory or early and late relapse was also comparable to what was reported in the literature (7) (9).

In the current study, the CR as the ORR in the GV regimen was 28.5%, which compares favorably with the COG phase II study of GV for children with R/R HL that reported a CR rate of 24% (30). Our results also documented the efficacy of the ICE regimen as 50.6% achieved CR, and 3.7% had PR, with an ORR of 54.3% at the time of consolidation. The published pediatric literature reported on the ICE response as a salvage regimen for pediatric R/R CHL are few; however, it was 33% as an ORR (CR + PR) in a study reported by Metzger et al. (9), and from 60% to 88% in other studies. These variations potentially were biased by the intensity of frontline therapy, timing of relapse, scheduling, and doses of ICE therapy, tools used in response assessment, and inclusion of adult patients in some studies (31) (32). In our results, it is obvious that the CR status at early evaluation and the ORR before consolidation were superior for the ICE regimen in comparison to the GV. Many factors were found to be significant for response by univariate analysis; however, aside from regimen, time to relapse and B-symptoms were significant in multivariate analysis. All these factors suggest that the response to salvage chemotherapy is highly influenced by the specific prognostic factors and tumor biology of CHL.

In the current study, the ICE regimen had a superior 3-year EFS in comparison to the GV; however, the 3-year OS was similar. Survival outcomes reported here were favorably similar to other children and adolescents series with R/R CHL (3) (9) (10) (33–35). 

The duration of the first remission had a significant impact on the response and outcome. Children with refractory disease to the first line had significantly lower response rates when compared to those who relapsed later. In our analysis, only 10.3% of the patients with refractory disease achieved CR to SDCT, compared to 50% and 55.8% for those with early and late relapse, respectively. Also, the 3-year EFS was 6.5% ± 8.6%, 40.7% ± 20%, and 51% ± 11.6%, for refractory, early, and late relapsed patients, respectively (P = 0.011), confirming the poor response and survival for patients with refractory disease. This finding is in agreement with previous pediatric studies with relapsed HL indicating the prognostic significance of the length of time between primary diagnosis and treatment failure (7) (10) (30) (34).

Achieving complete metabolic remission (CMR) with a Deauville score of 1–3 on salvage SDCT is highly predictive for outcome. The best time point for FDG-PET response assessment is not clearly defined, but is it generally done after two cycles of SDCT. Failure to demonstrate an improvement (less than CR/PR) should prompt consideration of a switch to an alternative regimen. A third cycle may be considered in slow responders (PR) to try to achieve CR (36). In contrast, the EuroNet Pediatric Hodgkin Lymphoma Group recommends switching to second-line SDCT if failed to achieve CMR after two cycles of salvage chemotherapy (1). Similarly, in our analysis, only 26.3% of patients who had PR on early evaluation could achieve CR before consolidation. Furthermore, the 3-year EFS was 62.7% ± 12.5% of patients who achieved CR on early evaluation versus 21.9% ± 11%, who had PR and the 3-year OS was 94% ± 6.7% for patients who achieved CR/PR at early evaluation vs. 56% ± 12%, for PD. Obviously, patients with recurrent disease that is unresponsive to conventional SDCT have a poor prognosis.

Many other poor prognostic factors at relapse were reported in our study as well as others such as B-symptoms, female sex, older age, higher-stage, elevated ESR, leukocytes, and anemia (1) (7) (24) (37–39), (40), (41).

In the current study, patients who did not receive radiotherapy during first line had a lower response rate, and inferior EFS and OS, but this was confounded by the fact that patients who did not receive radiotherapy had worse prognostic factors as a higher percentage had PD during first line (42.5%), B-symptoms (57.6%), ESR ≥50, mm/h (70%), and hemoglobin <10.5 gm/dl (13%) versus (9%), (44%), (53.6%), and (6.4%), respectively, for patients who received radiotherapy.

Of interest in our study, despite the better response rates to the ICE, versus the GV regimen, the 3-year OS was not statistically significant between both regimens because a higher number of patients in the GV group could be salvaged and further achieved CR (46.9%) on third lines when compared to the ICE group (27.3%). Furthermore, patients who failed the third line in the GV group received further fourth and fifth lines more than the ICE group with a higher percentage of them who underwent ASCT (P = 0.004). Also, the time era was earlier when the ICE was given compared to the GV and the follow-up time for the ICE group was lengthier (3.8 years) than the GV (2.6 years). The efficacy of immune and targeted therapy for R/R patients is well documented (14); however, in our analysis, the use of brentuximab vedotin as a single agent or in combination was more commonly used for subsequent relapse in recent years with an overall CR rate of 40%.

Our study is not designed to test the efficacy of the condition pretransplant preparative regimen; however, in our whole cohort of both groups, 51 patients received BEAM, and 31 received the CMV regimen before stem cell rescue. Forty-three (84%) maintained their CR after ASCT after BEAM versus (74%) after the CMV preparative regimen. This could also explain the higher OS rates for the GV group; however, it should be noticed that the BEAM regimen was used recently and longer follow-up is needed to evaluate the remission status after ASCT as the relatively slow-progressing nature of HL warrants a much longer follow-up.

The GV was well tolerated with fewer toxicities as compared to the ICE regimen. Myelosuppression, grade III-IV hematological toxicities, transfusion support, infectious complications, grade III electrolytes disturbance secondary to ifosfamide, carboplatin renal affection, and hemorrhagic cystitis were significantly higher in the ICE group. Due to the predominance of all these toxicities, the hospital admissions for supportive care mainly with FN were higher in the ICE being 54.2% compared with 2.1% of the GV cycles, adding much burden and cost to the healthcare providers.

The hepatotoxicity was not significantly different between both regimens except for grade III elevation of alanine aminotransferase, which was higher in the GV regimen; however, in all patients, it was transient and no delay or modification of chemotherapy was needed.

The reported pediatric mortality when reviewing the published literature from salvage therapy is low, as expected in the young age of the patients and lack of comorbidities. In our study, the treatment-related mortality was low in both groups and not significant when comparing both regimens.

Although the ICE regimen has higher CR rates in comparison to the GV, it is not optimal since it is associated with an increased risk of short- and long-term toxicities as treatment-related secondary malignant neoplasm associated with the use of alkylators and epipodophyllotoxins (42).

The retrospective nature of our study, some missing data, shorter follow-up duration in the GV group, and not addressing late toxicities are considered limitations in our study.

Finally, despite the very low toxicity profile of GV, it is not recommended due to the low response rate. Based on our results, a chemotherapy combination with higher efficacy than the GV regimen and lower toxicity than ICE is needed. Further studies on the alternative salvage second line, such as ifosfamide, gemcitabine, vinorelbine (IGEV), and prednisone (IGEV), for pediatric patients with relapsed or refractory CHL, is recommended.

## Data availability statement

The raw data supporting the conclusions of this article will be made available by the authors, without undue reservation.

## Ethics statement

The studies involving human participants were reviewed and approved by Institutional review board of National cancer institute, Cairo university, Egypt, Organization No. IORG0003381 IRP No. IRB00004025, Study ID. PO1910-30702, IRB Review. 20 October 2019, IRB Review type. Full Board Review, IRB Review action. Approved, IRB Review No. 201920009.3. Written informed consent to participate in this study was provided by the participants’ legal guardian/next of kin.

## Author contributions

AM did the data collection, wrote the primary draft, and revised the final draft with the authors. AH was involved in planning and supervision and aided in interpreting the results and worked on the manuscript, ME and AZ revised PET/CT scans for staging and response evaluation, EK revised pathology data, OH performed data analysis and statistical work. IS contributed to the drafting, interpreting the results, and critical revision and helped in the writing of the manuscript. All authors discussed the results and approved the final manuscript.

## References

[1] Daw S Hasenclever D Mascarin M Fernández-Teijeiro A Balwierz W Beishuizen A. Risk and response adapted treatment guidelines for managing first relapsed and refractory classical hodgkin lymphoma in children and young people. Recommendations from the EuroNet pediatric hodgkin lymphoma group. HemaSphere (2020) 4(1):e329. doi: 10.1097/HS9.0000000000000329 32072145 PMC7000476

[2] Smith CM Friedman DL. Advances in Hodgkin lymphoma: including the patient’s voice. Front Oncol (2022) 12(February):1–8. doi: 10.3389/fonc.2022.855725 PMC8914051 35280764

[3] Garaventa A Parodi S Guerrini G Farruggia P Sala A Pillon M. Outcome of children and adolescents with recurrent classical Hodgkin lymphoma: the Italian experience. Cancers (Basel). (2022) 14(6):1–16. doi: 10.3390/cancers14061471 PMC8946075 35326622

[4] Schmitz N Pfistner B Sextro M Sieber M Carella AM Haenel M. Aggressive conventional chemotherapy compared with high-dose chemotherapy with autologous haemopoietic stem-cell transplantation for relapsed chemosensitive hodgkin’s disease: a randomised trial. Lancet (2002) 359(9323):2065–71. doi: 10.1016/S0140-6736(02)08938-9 12086759

[5] Magagnoli M Balzarotti M Castagna L Demarco M Santoro A. What is the best option to cure patients with Resistant/Relapsing hodgkins disease? Curr Stem Cell Res Ther (2012) 1(3):419–24. doi: 10.2174/157488806778226786 18220885

[6] Bröckelmann PJ von Tresckow B. Risk stratification and prognostic biomarkers in relapsed Hodgkin lymphoma. Br J Haematol (2020) 190(6):813–4. doi: 10.1111/bjh.16844 32506494

[7] Schellong G Dörffel W Claviez A Körholz D Mann G Scheel-Walter HG. Salvage therapy of progressive and recurrent hodgkin’s disease: results from a multicenter study of the pediatric DAL/GPOH-HD study group. J Clin Oncol (2005) 23(25):6181–9. doi: 10.1200/JCO.2005.07.930 16135485

[8] Friedmann AM Wolfson JA Hudson MM Weinstein HJ Link MP Billett A. Relapse after treatment of pediatric hodgkin lymphoma: outcome and role of surveillance after end of therapy. Pediatr Blood Cancer (2013) 60(9):1458–63. doi: 10.1002/pbc.24568 PMC4313350 23677874

[9] Metzger ML Hudson MM Krasin MJ Wu J Kaste SC Kun LE. Initial response to salvage therapy determines prognosis in relapsed pediatric hodgkin lymphoma patients. Cancer (2010) 116(18):4376–84. doi: 10.1002/cncr.25225 PMC2936658 20564743

[10] Gorde-Grosjean S Oberlin O Leblanc T Pacquement H Donadieu J Lambilliotte A. Outcome of children and adolescents with recurrent/refractory classical Hodgkin lymphoma, a study from the société française de lutte contre le cancer des enfants et des adolescents (SFCE). Br J Haematol (2012) 158(5):649–56. doi: 10.1111/j.1365-2141.2012.09199.x 22757721

[11] Claviez A Kabisch H Suttorp M Peters C Hero B Schiller I. The impact of disease status at transplant and time to first relapse on outcome in children and adolescents with hodgkin’s lymphoma undergoing autologous stem cell transplantation. Blood (2004) 104(11). doi: 10.1182/blood.V104.11.1878.1878

[12] Castellino SM Pei Q Parsons SK Hodgson D McCarten K Horton T. Brentuximab vedotin with chemotherapy in pediatric high-risk hodgkin’s lymphoma. N Engl J Med (2022) 387(18):1649–60. doi: 10.1056/NEJMoa2206660 PMC9945772 36322844

[13] Castellino SM Pei Q Parsons SK Hodgson DC McCarten K Punnett A. Brentuximab vedotin and association with event-free survival (EFS) in children with newly diagnosed high-risk Hodgkin lymphoma (HL): a report from the children’s oncology group phase 3 study AHOD1331 (NCT 02166463). J Clin Oncol (2022) 40(16_suppl):7504–7504. doi: 10.1200/JCO.2022.40.16_suppl.7504

[14] Massano D Carraro E Mussolin L Buffardi S Barat V Zama D. Brentuximab vedotin in the treatment of paediatric patients with relapsed or refractory hodgkin’s lymphoma: results of a real-life study. Pediatr Blood Cancer (2022) 69(10):e29801. doi: 10.1002/pbc.29801 35656841

[15] Moskowitz AJ Schöder H Yahalom J McCall SJ Fox SY Gerecitano J. PET-adapted sequential salvage therapy with brentuximab vedotin followed by augmented ifosamide, carboplatin, and etoposide for patients with relapsed and refractory hodgkin’s lymphoma: a non-randomised, open-label, single-centre, phase 2 study. Lancet Oncol (2015) 16(3):284–92. doi: 10.1016/S1470-2045(15)70013-6 25683846

[16] Herrera AF Moskowitz AJ Bartlett NL Vose JM Ramchandren R Feldman TA. Interim results of brentuximab vedotin in combination with nivolumab in patients with relapsed or refractory Hodgkin lymphoma. Blood (2018) 131(11):1183–94. doi: 10.1182/blood-2017-10-811224 PMC5855021 29229594

[17] Moskowitz AJ Shah G Schöder H Ganesan N Drill E Hancock H. Phase II trial of pembrolizumab plus gemcitabine, vinorelbine, and liposomal doxorubicin as second-line therapy for relapsed or refractory classical Hodgkin lymphoma. J Clin Oncol (2021) 39(28):3109–17. doi: 10.1200/JCO.21.01056 PMC9851707 34170745

[18] Daw S Wynn R Wallace H. Management of relapsed and refractory classical Hodgkin lymphoma in children and adolescents. Br J Haematol (2011) 152(3):249–60. doi: 10.1111/j.1365-2141.2010.08455.x 21133886

[19] Swerdlow SH Campo E Pileri SA Lee Harris N Stein H Siebert R. The 2016 revision of the world health organization classification of lymphoid neoplasms. Blood (2016) 127(20):2375–90. doi: 10.1182/blood-2016-01-643569 PMC4874220 26980727

[20] Josting A Rueffer U Franklin J Sieber M Diehl V Engert A. Prognostic factors and treatment outcome in primary progressive Hodgkin lymphoma: a report from the German Hodgkin lymphoma study group. Blood (2000) 96(4):1280–6. doi: 10.1182/blood.V96.4.1280.h8001280_1280_1286 10942369

[21] Cheson BD Fisher RI Barrington SF Cavalli F Schwartz LH Zucca E. Recommendations for initial evaluation, staging, and response assessment of hodgkin and non-hodgkin lymphoma: the lugano classification. J Clin Oncol (2014) 32(27):3059–67. doi: 10.1200/JCO.2013.54.8800 PMC4979083 25113753

[22] National Cancer Institute. Common terminology criteria for adverse events (CTCAE) common terminology criteria for adverse events (CTCAE) v5.0. National Cancer Institute (2017).

[23] Smith RS Chen Q Hudson MM Link MP Kun L Weinstein H. Prognostic factors for children with hodgkin’s disease treated with combined-modality therapy. J Clin Oncol (2003) 21(10):2026–33. doi: 10.1200/JCO.2003.07.124 12743158

[24] Josting A Franklin J May M Koch P Beykirch MK Heinz J. New prognostic score based on treatment outcome of patients with relapsed hodgkin’s lymphoma registered in the database of the German hodgkin’s lymphoma study group. J Clin Oncol (2002) 20(1):221–30. doi: 10.1200/JCO.2002.20.1.221 11773173

[25] Stel VS Dekker FW Tripepi G Zoccali C Jager KJ. Survival analysis I: the Kaplan-Meier method. Nephron - Clin Pract (2011) 119(1):c83–8. doi: 10.1159/000324758 21677442

[26] Bender R Augustin T Blettner M. Generating survival times to simulate cox proportional hazards models. Stat Med (2005) 24(11):1713–23. doi: 10.1002/sim.2059 15724232

[27] Kelly KM. Hodgkin Lymphoma in children and adolescents: improving the therapeutic index. Hematol (United States). (2015) 2015(1):514–21. doi: 10.1182/blood-2015-07-641035 26637767

[28] Alkhayat N Alshahrani M Elyamany G Sedick Q Ibrahim W Hamzi H. Clinicopathologic features and therapy outcome in childhood hodgkin’s lymphoma: a report from tertiary care center in Saudi Arabia. J Egypt Natl Canc Inst (2021) 33(1):21. doi: 10.1186/s43046-021-00078-0 34396456 PMC13316896

[29] Aquino S Clavio M Rossi E Vignolo L Miglino M Spriano M. Therapy of hodgkin’s lymphoma in clinical practice: a retrospective long-term follow-up analysis. Oncol Lett (2011) 2(2):289–95. doi: 10.3892/ol.2011.255 PMC3410564 22866079

[30] Cole PD Schwartz CL Drachtman RA De Alarcon PA Chen L Trippett TM. Phase II study of weekly gemcitabine and vinorelbine for children with recurrent or refractory hodgkin’s disease: a children’s oncology group report. J Clin Oncol (2009) 27(9):1456–61. doi: 10.1200/JCO.2008.20.3778 PMC2668553 19224841

[31] Moskowitz CH Nimer SD Zelenetz AD Trippett T Hedrick EE Filippa DA. A 2-step comprehensive high-dose chemoradiotherapy second-line program for relapsed and refractory Hodgkin disease: analysis by intent to treat and development of a prognostic model. Blood (2001) 97(3):616–23. doi: 10.1182/blood.V97.3.616 11157476

[32] Karuturi M Younes A Fayad L Kwak L Pro B Shah J. Ifosfamide, carboplatin, etoposide with or without bortezomib in patients with relapsed/refractory Hodgkin lymphoma: results of a randomized phase II trial. Leuk Lymph (2016) 57(2):445–7. doi: 10.3109/10428194.2015.1032966 PMC5215977 25804933

[33] Claviez A Canals C Dierickx D Stein J Badell I Pession A. Allogeneic hematopoietic stem cell transplantation in children and adolescents with recurrent and refractory Hodgkin lymphoma: an analysis of the European group for blood and marrow transplantation. Blood (2009) 114(10):2060–7. doi: 10.1182/blood-2008-11-189399 19498021

[34] Shankar A Hayward J Kirkwood A Mccarthy K Hewitt M Morland B. Treatment outcome in children and adolescents with relapsed Hodgkin lymphoma - results of the UK HD3 relapse treatment strategy. Br J Haematol (2014) 165(4):534–44. doi: 10.1111/bjh.12768 24754633

[35] Talleur AC Flerlage JE Shook DR Chilsen AM Hudson MM Cheng C. Autologous hematopoietic cell transplantation for the treatment of relapsed/refractory pediatric, adolescent, and young adult Hodgkin lymphoma: a single institutional experience. Bone Marrow Transplant [Internet] (2020) 55(7):1357–66. doi: 10.1038/s41409-020-0879-4 32273588

[36] Collins GP Parker AN Pocock C Kayani I Sureda A Illidge T. Guideline on the management of primary resistant and relapsed classical Hodgkin lymphoma. Br J Haematol (2014) 164(1):39–52. doi: 10.1111/bjh.12582 24117159

[37] Akhtar S El Weshi A Rahal M Abdelsalam M Al Husseini H Maghfoor I. High-dose chemotherapy and autologous stem cell transplant in adolescent patients with relapsed or refractory hodgkin’s lymphoma. Bone Marrow Transplant (2010) 45(3):476–82. doi: 10.1038/bmt.2009.197 19734949

[38] Shah GL Moskowitz CH. Transplant strategies in relapsed/refractory Hodgkin lymphoma. Blood (2018) 131(15):1689–97. doi: 10.1182/blood-2017-09-772673 PMC5897866 29500170

[39] Lieskovsky YYE Donaldson SS Torres MA Wong RM Amylon MD Link MP. High-dose therapy and autologous hematopoietic stem-cell transplantation for recurrent or refractory pediatric hodgkin’s disease: results and prognostic indices. J Clin Oncol (2004) 22(22):4532–40. doi: 10.1200/JCO.2004.02.121 15542804

[40] Harker-Murray PD Drachtman RA Hodgson DC Chauvenet AR Kelly KM Cole PD. Stratification of treatment intensity in relapsed pediatric Hodgkin lymphoma. Pediatr Blood Cancer (2014) 61(4):579–86. doi: 10.1002/pbc.24851 24504790

[41] Wolden SL Chen L Kelly KM Herzog P Gilchrist GS Thomson J. Long-term results of CCG 5942: a randomized comparison of chemotherapy with and without radiotherapy for children with hodgkin’s lymphoma - a report from the children’s oncology group. J Clin Oncol (2012) 30(26):3174–80. doi: 10.1200/JCO.2011.41.1819 PMC3434976 22649136

[42] Suyanı E Sucak GT Akı ŞZ Yeğin ZA Özkurt ZN Yağcı M. Gemcitabine and vinorelbine combination is effective in both as a salvage and mobilization regimen in relapsed or refractory Hodgkin lymphoma prior to ASCT. Ann Hematol (2011) 90(6):685–91. doi: 10.1007/s00277-010-1113-z 21072518

